# Expeditious Entry to Novel 2-Methylene-2,3-dihydrofuro[3,2-*c*]chromen-2-ones from 6-Chloro-4-hydroxychromen-2-one and Propargylic Alcohols

**DOI:** 10.3390/molecules16086470

**Published:** 2011-08-02

**Authors:** Noel Nebra, Alba E. Díaz-Álvarez, Josefina Díez, Victorio Cadierno

**Affiliations:** Departamento de Química Orgánica e Inorgánica, IUQOEM-CSIC, Facultad de Química, Universidad de Oviedo, E-33071 Oviedo, Spain

**Keywords:** chromen-2-ones, furochromen-2-ones, propargylic alcohols, ruthenium catalysts, propargylation, cycloisomerization

## Abstract

A catalytic system consisting of the ruthenium(II) complex [Ru(*η*^3^-2-C_3_H_4_Me)(CO)(dppf)][SbF_6_] (dppf = 1,1’-bis(diphenylphosphino)ferrocene) and trifluoroacetic acid has been used to promote the coupling of secondary propargylic alcohols with 6-chloro-4-hydroxychromen-2-one. The reactions afforded unusual 2-methylene-2,3-dihydrofuro[3,2-*c*]chromen-2-ones in good yields.

## 1. Introduction

**Figure 1:**
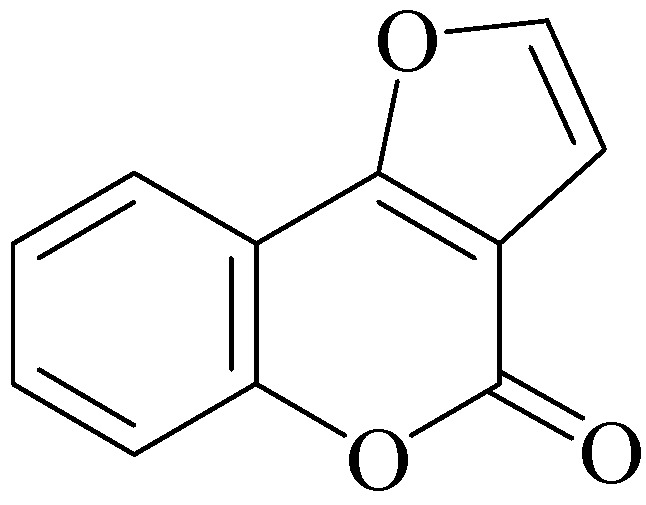
The furo[3,2-*c*]chromen-2-one skeleton.

Chromen-2-ones (coumarins) constitute an important family of heterocyclic compounds of natural origin which have attracted considerable attention for many years due to their versatile applications [1,2,3,4]. Among them, furochromen-2-ones (furocoumarins), tricyclic systems in which a furan ring is fused to the chromen-2-one unit, are of particular interest since they exhibit potent biological and pharmacological activity [5,6,7,8]. Although several methods of synthesis are presently known [7,8], new approaches for the rapid and selective construction of furochromen-2-one scaffolds are still highly desirable. In this context, recent efforts by different groups have been focused in the *one-pot* synthesis of furo[3,2-*c*]chromen-2-ones (Figure 1) from readily available starting materials, with successful examples including CAN-mediated cycloaddition of 4-hydroxychromen-2-one with terminal alkynes [9,10], rhodium(II)-catalyzed heterocyclization of 3-diazo-2,4-chromenediones with terminal alkynes [11,12], cascade addition/cyclization/oxidation of 3-alkynyl-chromones [13,14], Sonogashira-acetylide coupling/demethylation/cyclization of 3-iodo-4-methoxychromen-2-ones [15,16] and alkynylation/hydroalkoxylation of 3-bromo-4-acetoxychromen-2-ones [17].

**Scheme 1:**
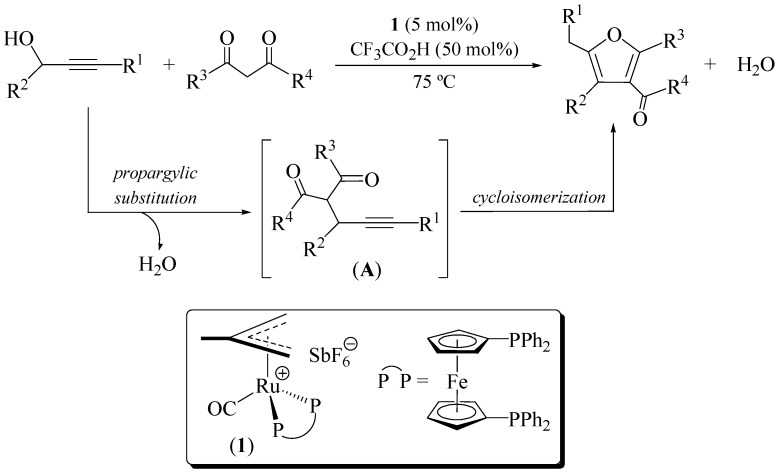
Direct synthesis of furans from alkynols and 1,3-dicarbonyl compounds.

In the course of our studies focused on the application of ruthenium catalysts for the construction of furan- and pyrrole-ring frameworks [18,19,20,21,22,23,24,25], we disclosed a straightforward approach of tetra-substituted furans from readily accessible secondary propargylic alcohols and 1,3-dicarbonyl compounds (Scheme 1) [18]. The process, which proceeds in a *one-pot* manner, involves the initial trifluoroacetic acid-promoted propargylation of the 1,3-dicarbonyl compound, and subsequent cycloisomerization of the resulting *γ*-ketoalkyne **A** (5-*exo* cyclization + aromatization) catalyzed by the 16-electron allyl-ruthenium(II) complex [Ru(*η*^3^-2-C_3_H_4_Me)(CO)(dppf)][SbF_6_] (**1**).

By applying this synthetic route a large variety of furans containing carbonyl functionalities on the aromatic ring, could be prepared in good yields starting from both terminal and internal secondary alkynols, and β-diketones or β-keto esters [18,21]. In addition, we also demonstrated that furo[3,2-*c*]chromen-2-ones are also accessible by this route using 4-hydroxychromen-2-one as substrate, representing an appealing *one-pot* method of synthesis for this type of heterocycles [21]. Related work by Zhou and co-workers also confirmed the utility of this propargylation-cycloisomerization sequence for the construction of furochromen-2-one skeletons [26].

**Figure 2:**
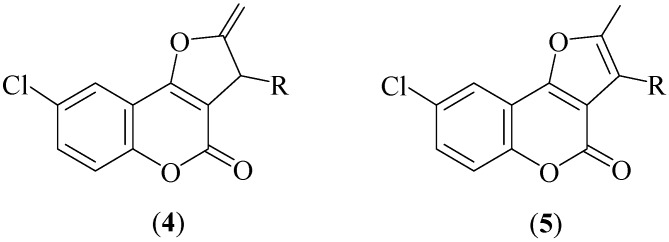
Structures of compounds **4** and **5**.

Following with these studies, herein we would like to communicate that related C–C coupling processes involving 6-chloro-4-hydroxychromen-2-one and terminal propargylic alcohols HC≡CC(OH)HR result in the selective formation of the 2-methylene-2,3-dihydrofuro[3,2-*c*]chromen-2-one derivatives **4** (Figure 2), instead of the expected 8-chloro-substituted furo[3,2-*c*]chromen-2-ones **5**, due to the reluctance of the former to undergo aromatization of the five-membered ring.

## 2. Results and Discussion

**Scheme 2:**
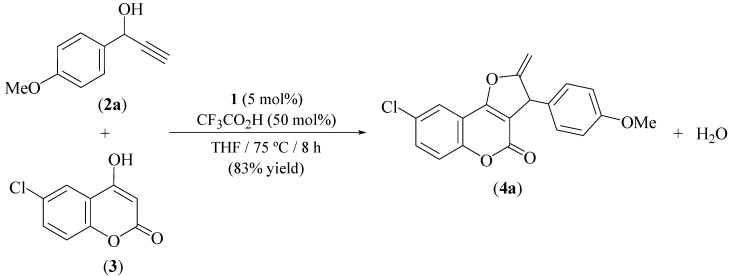
Catalytic synthesis of compound **4a** under classical thermal conditions.

Initially, the coupling of the secondary propargylic alcohol 1-(4-methoxyphenyl)-2-propyn-1-ol (**2a**) with 6-chloro-4-hydroxychromen-2-one (**3**) was investigated under the same reaction conditions previously employed by us in the synthesis of furo[3,2-*c*]chromen-2-one derivatives starting from 4-hydroxychromen-2-one [21], that is, heating a THF solution of both reactants (equimolar mixture) at 75 *°*C in the presence of 50 mol% of trifluoroacetic acid and 5 mol% of the allyl-ruthenium(II) complex **1** (Scheme 2). Almost complete disappearance of the starting materials, accompanied by the selective formation of a single reaction product **4a**, was observed by GC after 8 hours of heating.

**Figure 3:**
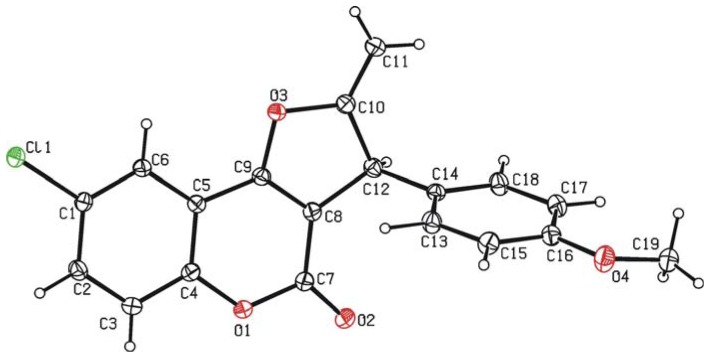
ORTEP-type view of the structure of compound **4a** showing the crystallographic labelling scheme. Thermal ellipsoids are drawn at the 20% probability level.

Appropriate chromatographic workup allowed the isolation of **4a** as a crystalline yellow solid in 83% yield. NMR spectroscopic data obtained for **4a** clearly revealed the selective formation of a 2-methylene-2,3-dihydrofuran unit, instead of the expected aromatic 2-methylfuran one (details are given in the Experimental), a fact that was unambiguously confirmed by means of a single-crystal X-ray diffraction study (an ORTEP view of the molecule is shown in Figure 3; selected bonding parameters are listed in Table 2). The bond distance C10-C11 (1.321(3) Å) showed the expected value for a C=C bond, while that observed for C10-C12 (1.523(3) Å) falls within the expected range for a C(sp^2^)-C(sp^3^) single bond.

**Table 2:** Selected bond distances (Å) and angles (°) for compound **4a**.

**Distances**	
C8-C9	1.338(3)
C9-O3	1.360(2)
O3-C10	1.417(2)
C10-C11	1.321(3)
C10-C12	1.523(3)
C12-C8	1.507(3)
C7-O1	1.393(3)
C7-O2	1.211(3)
C1-Cl1	1.738(2)
**Angles**	
C8-C9-O3	114.13(18)
C9-O3-C10	106.39(16)
O3-C10-C11	118.8(2)
C11-C10-C12	131.1(2)
O3-C10-C12	110.07(16)
C10-C12-C8	99.46(17)
C12-C8-C9	109.77(18)

**Scheme 3:**
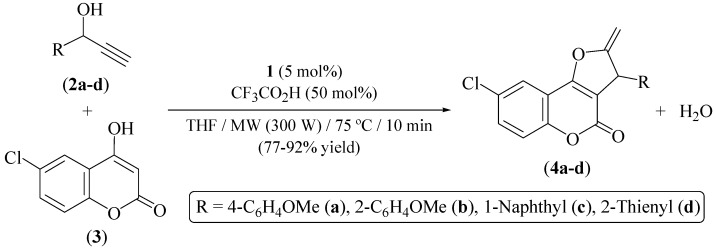
Catalytic synthesis of compound **4a–d** under MW-irradiation.

The use of microwave (MW) irradiation represents a convenient alternative to the conventional thermal heating in organic synthesis since a more effective energy transfer to the system takes place, thus shortening considerably the reaction times and improving in many cases the product yields [27,28,29]. Accordingly, we have found that, performing the same coupling reaction of alkynol **2a** with **3** under controlled microwave heating at 75 *°*C, selective and almost quantitative formation of 2-methylene-2,3-dihydrofuro[3,2-*c*]chromen-2-one (**4a**, 99% GC-yield; 89% isolated yield) takes place after only 10 min. As shown in Scheme 3, the process is general since the related heterocycles **4b–d** could also be synthesized in good yields (77%–92%) by reacting **3** with the secondary propargylic alcohols 1-(2-methoxyphenyl)-2-propyn-1-ol (**2b**), 1-(1-naphthyl)-2-propyn-1-ol (**2c**) and 1-(2-thienyl)-2-propyn-1-ol (**2d**) under the same MW conditions.

The presence of a 2-methylene-2,3-dihydrofuran moiety in the structure of these compounds was readily identified by the appearance of a high-field CH carbon resonance at 43–49 ppm (CHR unit) and a CH_2_ signal at *ca.* 92 ppm, typical of a terminal olefinic =CH_2_ unit, in their ^13^C{^1^H}-NMR spectra (DEPT experiments). Characteristic ^1^H-NMR peaks for these units were also observed at 4.5–5.5 ppm (details can be found in the Experimental).

**Scheme 4:**
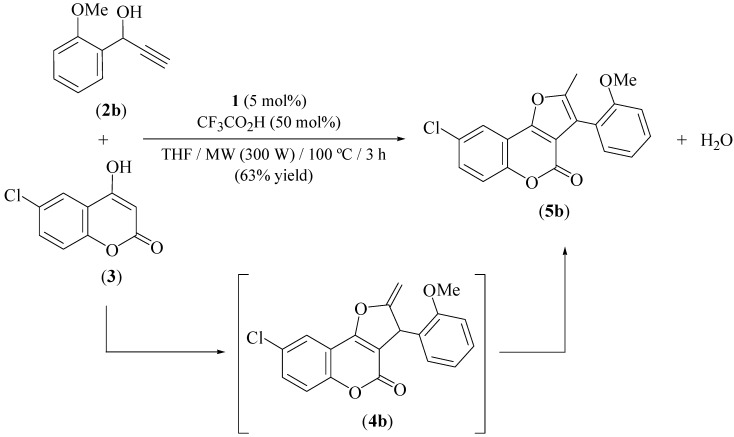
Synthesis of the furo[3,2-*c*]chromen-2-one **5b**.

At this point, it is worthy of note that occurrence of 2-methylene-2,3-dihydrofuro[3,2-*c*]chromen-2-ones has been scarcely documented in the literature [30,31,32,33,34,35], with most of the know examples being disubstituted at the C-3 position of the 2-methylene-2,3-dihydrofuran ring which prevents their tautomerization into the corresponding 2-methyl-furo[3,2-*c*]chromen-2-ones. In this sense, the reluctance shown by compounds **4a–d** to aromatize under the acidic conditions employed merits to be highlighted. In fact, only in the case of **4b** such aromatization process could be observed after prolonged MW irradiation (3 h) of the reaction mixture at 100 *°*C. Under this conditions, the novel 8-chloro-substituted furo[3,2-*c*]chromen-2-one **5b** could be synthesized with an acceptable 63% yield and fully characterized (Scheme 4).

## 3. Experimental

### 3.1. General

Solvents were dried by standard methods and distilled under nitrogen before use. The complex [Ru(η^3^-2-C_3_H_4_Me)(CO)(dppf)][SbF_6_] (**1**) [36] and propargylic alcohols **2a–d** [37] were prepared by following the methods reported in the literature. Flash chromatography was performed using Merck silica gel 60 (230–400 mesh). Melting points were determined in a Gallenkamp apparatus and are uncorrected. Infrared spectra were recorded on a Perkin-Elmer 1720-XFT spectrometer. NMR spectra were recorded on a Bruker DPX-300 instrument at 300 MHz (^1^H) or 75.4 MHz (^13^C). The chemical shift values (*δ*) are given in parts per million and are referred to the residual peak of the deuterated solvent used (CDCl_3_). High-resolution mass spectra (HRMS) were provided by the Mass Spectrometry Service of the Instituto de Investigaciones Químicas (IIQ-CSIC, Seville). CCDC 831021 contains the supplementary crystallographic data for this paper. These data can be obtained free of charge from The Cambridge Crystallographic Data Centre via www.ccdc.cam.ac.uk/data_request/cif.

### 3.2. Synthesis of the 8-chloro-2-methylene-2,3-dihydrofuro[3,2-c]chromen-2-ones ***4a–d***

Under a nitrogen atmosphere, a pressure-resistant septum-sealed glass vial was charged with the corresponding propargylic alcohol **2a–d** (1 mmol), 6-chloro-4-hydroxychromen-2-one (**3**) (0.197 g, 1 mmol), THF (0.5 mL), [Ru(*η*^3^-2-C_3_H_4_Me)(CO)(dppf)][SbF_6_] (**1**) (0.049 g, 0.05 mmol), CF_3_CO_2_H (37 µL, 0.5 mmol) and a magnetic stirring bar. The vial was then placed inside the cavity of a CEM Discover^®^ S-Class microwave synthesizer and power was held at 300 W until the desired temperature was reached (75 °C). Microwave power was automatically regulated for the remainder of the experiment (10 min) to maintain the temperature (monitored by a built-in infrared sensor). Then, the vial was cooled to room temperature, the volatiles removed under vacuum, and the residue purified by flash chromatography (silica gel) using a mixture EtOAc/hexanes (1:20) as eluent. Characterization data for the novel 8-chloro-2-methylene-2,3-dihydrofuro[3,2-*c*]chromen-2-ones **4a–d** are as follows:

*8-Chloro-3-(4-methoxyphenyl)-2-methylene-2,3-dihydrofuro[3,2-c]chromen-2-one* (**4a**). Yellow solid (0.303 g, 89%); m.p. 125–127 *°*C; IR (Nujol) *ν* = 1721 (C=O) cm^−1^; ^1^H-NMR (CDCl_3_) *δ* = 3.78 (s, 3H), 4.52 (dd, 1H, *J* = 3.4 and 2.8 Hz), 5.11 (m, 2H), 6.87 (d, 2H, *J* = 8.5 Hz), 7.23 (d, 2H, *J* = 8.5 Hz), 7.32 (d, 1H, *J* = 8.8 Hz), 7.53 (dd, 1H, *J* = 8.8 and 2.2 Hz), 7.75 (d, 1H, *J* = 2.2 Hz) ppm; ^13^C-NMR (CDCl_3_) *δ* = 47.7, 55.2, 91.7, 107.4, 112.5, 114.2, 118.5, 122.2, 128.8, 129.6, 131.2, 132.7, 153.5, 159.1, 157.8, 162.9, 164.8 ppm; HRMS (EI) *m/z* = 340.0501, C_19_H_13_O_4_Cl requires 340.0502.

*8-Chloro-3-(2-methoxyphenyl)-2-methylene-2,3-dihydrofuro[3,2-c]chromen-2-one* (**4b**). Yellow solid (0.262 g, 77%); m.p. 122–124 *°*C; IR (Nujol) *ν* = 1748 (C=O) cm^−1^; ^1^H-NMR (CDCl_3_) *δ* = 3.79 (s, 3H), 4.55 (dd, 1H, *J* = 3.0 and 2.5 Hz), 5.12 (m, 2H), 6.82–6.91 (m, 3H), 7.25 (m, 1H), 7.34 (d, 1H, *J* = 8.9 Hz), 7.55 (dd, 1H, *J* = 8.9 and 2.4 Hz), 7.76 (d, 1H, *J* = 2.2 Hz) ppm; ^13^C-NMR (CDCl_3_) *δ* = 48.5, 55.3, 92.1, 107.2, 112.6, 113.0, 113.8, 118.6, 120.1, 122.3, 129.8, 130.0, 132.9, 140.7, 153.6, 157.9, 160.0, 163.4, 164.3 ppm; HRMS (EI) *m/z* = 340.0513, C_19_H_13_O_4_Cl requires 340.0502.

*8-Chloro-3-(1-naphthyl)-2-methylene-2,3-dihydrofuro[3,2-c]chromen-2-one* (**4c**). Yellow solid (0.331 g, 92%); m.p. 140–142 *°*C; IR (Nujol) *ν* = 1719 (C=O) cm^−1^; ^1^H-NMR (CDCl_3_) *δ* = 4.55 (dd, 1H, *J* = 3.3 and 2.5 Hz), 5.15 (dd, 1H, *J* = 3.3 and 2.5 Hz), 5.33 (dd, 1H, *J* = 3.3 and 3.3 Hz), 7.33–7.49 (m, 5H), 7.81–7.85 (m, 5H) ppm; ^13^C-NMR (CDCl_3_) *δ* = 48.8, 92.3, 107.3, 112.6, 118.7, 122.4, 125.3, 126.2, 126.4, 127.0, 127.7, 127.9, 129.0, 129.8, 132.9, 133.0, 133.4, 136.5, 153.6, 157.9, 163.4, 164.5 ppm; HRMS (EI) *m/z* = 360.0557, C_22_H_13_O_3_Cl requires 360.0553.

*8-Chloro-3-(2-thienyl)-2-methylene-2,3-dihydrofuro[3,2-c]chromen-2-one* (**4d**). Orange solid (0.253 g, 80%); m.p. 131–133 *°*C; IR (Nujol) *ν* = 1733 (C=O) cm^−1^; ^1^H-NMR (CDCl_3_) *δ* = 4.72 (dd, 1H, *J* = 3.5 and 2.4 Hz), 5.18 (dd, 1H, *J* = 3.5 and 2.7 Hz), 5.48 (dd, 1H, *J* = 2.7 and 2.4 Hz), 6.98 (dd, 1H, *J* = 5.2 and 3.6 Hz), 7.12 (dd, 1H, *J* = 3.6 and 1.1 Hz), 7.25 (dd, 1H, *J* = 5.2 and 1.1 Hz), 7.35 (d, 1H, *J* = 9.0 Hz), 7.56 (dd, 1H, *J* = 9.0 and 2.5 Hz), 7.75 (d, 1H, *J* = 2.5 Hz) ppm; ^13^C-NMR (CDCl_3_) *δ* = 43.0, 92.3, 106.3, 112.1, 118.2, 122.0, 124.9, 125.6, 126.7, 129.4, 132.7, 141.2, 153.1, 157.3, 162.9, 163.0 ppm; HRMS (EI) *m/z* = 315.9971, C_16_H_9_O_3_ClS requires 315.9961.

### 3.3. Synthesis of 8-chloro-3-(2-methoxyphenyl)-2-methyl-furo[3,2-c]chromen-2-one *(**5b**)*

Under nitrogen atmosphere, a pressure-resistant septum-sealed glass vial was charged with 1-(2-methoxyphenyl)-2-propyn-1-ol (**2b**, 0.162 g, 1 mmol), 6-chloro-4-hydroxychromen-2-one (**3**, 0.197 g, 1 mmol), THF (0.5 mL), [Ru(*η*^3^-2-C_3_H_4_Me)(CO)(dppf)][SbF_6_] (**1**, 0.049 g, 0.05 mmol), CF_3_CO_2_H (37 µL, 0.5 mmol) and a magnetic stirring bar. The vial was then placed inside the cavity of a CEM Discover® S-Class microwave synthesizer and power was held at 300 W until the desired temperature was reached (100 *°*C). Microwave power was automatically regulated for the remainder of the experiment (3 h) to maintain the temperature (monitored by a built-in infrared sensor). Then, the vial was cooled to room temperature, the volatiles removed under vacuum, and the residue purified by flash chromatography (silica gel) using a mixture EtOAc/hexanes (1:20) as eluent to give **5b**. Yellow solid (0.214 g, 63%); m.p. 120–122 *°*C; IR (Nujol) *ν* = 1749 (C=O) cm^−1^; ^1^H-NMR (CDCl_3_) *δ* = 2.42 (s, 3H), 3.82 (s, 3H), 7.01–7.08 (m, 2H), 7.26–7.43 (m, 4H), 7.85 (d, 1H, *J* = 2.5 Hz) ppm; ^13^C-NMR (CDCl_3_) *δ* = 12.1, 55.1, 110.6, 113.7, 116.2, 118.0, 118.3, 119.6, 120.0, 129.2, 129.4, 131.0, 132.9, 136.5, 150.1, 152.5, 156.9, 162.5, 163.0 ppm; HRMS (EI) *m/z* = 340.0496, C_19_H_13_O_4_Cl requires 340.0502.

### 3.4. X-ray Crystal Structure Determination of Compound ***4a***

The most relevant crystal and refinement data are collected in Table 1. Data collection was performed on a Oxford Diffraction Xcalibur Nova single crystal diffractometer, using Cu-K*α* radiation. Images were collected at a 65 mm fixed crystal-to-detector distance using the oscillation method, with 1° oscillation and a 5 s exposure time per image. Data collection strategy was calculated with the program CrysAlis Pro CCD [38]. Data reduction and cell refinement were performed with the program CrysAlis Pro RED [38]. An empirical absorption correction was applied using the SCALE3 ABSPACK algorithm as implemented in the program CrysAlis Pro RED [38]. The software package WinGX was used for space group determination, structure solution and refinement [39]. The structure was solved by direct methods using SIR92 [40]. Isotropic least-squares refinement on *F*^2^ using SHELXL97 was performed [41]. During the final stages of the refinements, all the positional parameters and the anisotropic temperature factors of all the non-H atoms were refined. The coordinates of the H atoms were found from different Fourier maps and included in a refinement with isotropic parameters. The function minimized was [Σ*wF*o^2^ − *F*c^2^)/Σ*w*(*F*o^2^)]^1/2^ where *w* = 1/[*σ*^2^(*F*o^2^) + (a*P*)^2^ + b*P*] (a = 0.0902; b = 0.0000) with *σ*^2^(*F*o^2^) from counting statistics and *P* = (Max (*F*o^2^ + 2*F*c^2^)/3. Atomic scattering factors were taken from the International Tables for X-ray Crystallography [42]. The crystallographic plot was made with PLATON [43].

**Table 1:** Crystal data and structure refinement parameters for compound **4a**.

Empirical formula	C_19_H_13_O_4_Cl
Formula weight	340.74
Temperature	150(2) K
Wavelength	1.5418 Å
Crystal system, space group	*triclinic*, *P*-1
Unit cell dimensions	*a* = 4.8366(2) Å *α* = 94.822(4)°
	*b* = 11.0016(5) Å *β* = 90.363(4)°
	*c* = 14.6466(7) Å *γ* = 94.200(4)°
Volume	774.45(6) Å^3^
*Z*, calculated density	2, 1.461 mg/m^3^
Absorption coefficient	2.369 mm^−1^
*F*(000)	352
Crystal size	0.37 × 0.03 × 0.02 mm
Theta range for data collection	3.03 to 73.76°
Limiting indices	−4 ≤ h ≤ 6, −13 ≤ k ≤ 12, −17 ≤ l ≤ 17
Reflections collected / unique	7403/2919 (*R_int_* = 0.0214)
Completeness to theta = 73.76º	93.4%
Refinement method	Full-matrix least-squares on *F*^2^
Data / restrains / parameters	2919/0/269
Goodness-of-fit on *F*^2^	1.166
Final *R* indices [*I* > 2sigma(*I*)]	*R*_1_ = 0.0411, *wR*_2_ = 0.1177
*R* indices (all data)	*R*_1_ = 0.0517, *wR*_2_ = 0.1354
Largest diff. peak and hole	0.334 and −0.267 e∙Å^3^

## 4. Conclusions

In summary, an efficient synthesis of unusual and remarkably stable 2-methylene-2,3-dihydrofuro[3,2-*c*]chromen-2-one derivatives, by coupling of secondary propargylic alcohols with commercially available 6-chloro-4-hydroxychromen-2-one, has been developed with the aid of the catalytic system [Ru(*η*^3^-2-C_3_H_4_Me)(CO)(dppf)][SbF_6_]/CF_3_CO_2_H. Apparently, the presence of the electron-withdrawing Cl substituent on the 4-hydroxychromen-2-one skeleton exerts a marked influence on the behavior of these species since, as previously described by us [21], the same reactions performed with its non-substituted counterpart leads to the selective formation of isomeric furo[3,2-*c*]chromen-2-ones by aromatization of the five-membered ring. Overall, the results reported herein represent a new example of the utility of the allyl-ruthenium(II) complex **1** in synthetic organic chemistry [25].
